# Co-Circulation of 2 Oropouche Virus Lineages, Amazon Basin, Colombia, 2024

**DOI:** 10.3201/eid3011.240405

**Published:** 2024-11

**Authors:** Jaime Usuga, Daniel Limonta, Laura S. Perez-Restrepo, Karl A. Ciuoderis, Isabel Moreno, Angela Arevalo, Vanessa Vargas, Michael G. Berg, Gavin A. Cloherty, Juan P. Hernandez-Ortiz, Jorge E. Osorio

**Affiliations:** National University of Colombia, Medellin, Colombia (J. Usuga, L.S. Perez-Restrepo, K.A. Ciuoderis, I. Moreno, A. Arevalo, V. Vargas, J.P. Hernandez-Ortiz, J.E. Osorio); University of Wisconsin, Madison, Wisconsin, USA (D. Limonta, K.A. Ciuoderis, J.E. Osorio); Abbott Diagnostics/Abbott Pandemic Defense Coalition, Abbott Park, Illinois, USA (M.G. Berg, G.A. Cloherty)

**Keywords:** Oropouche virus, reassortment, fever, sequencing, outbreak, lineages, reassortant, vector-borne infections, viruses, zoonoses, Leticia, Amazon Basin, Colombia

## Abstract

In early 2024, explosive outbreaks of Oropouche virus (OROV) linked to a novel lineage were documented in the Amazon Region of Brazil. We report the introduction of this lineage into Colombia and its co-circulation with another OROV lineage. Continued surveillance is needed to prevent further spread of OROV in the Americas.

Oropouche virus (OROV; *Orthobunyavirus oropoucheense*) is a reemerging arbovirus belonging to the family Peribunyaviridae. The large (L), medium (M), and small (S) single-stranded, negative-sense RNA segments of the OROV genome are susceptible to reassortments (*1*). Besides acute fever, OROV can cause meningitis and encephalitis. Although *Culicoides paraensis* biting midges are the main vector for OROV in urban cycles, different insect species are vectors in sylvatic cycles. Vertebrates, such as primates, small mammals, and possibly birds, are reservoir hosts (*1*,*2*).

OROV has spread rapidly in South America, causing >500,000 human infections (*2*,*3*). Most OROV cases have occurred in the Amazon in Brazil, where explosive outbreaks, sometimes affecting thousands of inhabitants, have been reported since OROV was identified in Trinidad and Tobago in 1955 (*2*,*4*,*5*). During 2019–2021, OROV was responsible for >10% and dengue virus (DENV) for 20% of acute febrile illness cases at 4 sites in Colombia: Cúcuta, Cali, Villavicencio, and Leticia (*6*). Phylogenetic analysis revealed 2 separate OROV introductions into those sites in Colombia from bordering Ecuador or Peru (*6*).

In early February 2024, the Pan American Health Organization and World Health Organization issued an epidemiologic alert because of the dramatic increase in OROV cases in 4 states within the Amazon Region of Brazil (*7*). The state of Amazonas, Brazil, alone had >1,000 quantitative reverse transcription PCR (qRT-PCR)–confirmed cases reported during 2023–January 2024 (*8*). After analyzing hundreds of full-length genomes isolated during the large-scale OROV outbreak in Brazil, multiple reassortment events were identified, indicating a new OROV lineage, BR-2015-2024 (*9*; F.C.M. Iani et al., unpub. data, https://doi.org/10.1101/2024.08.02.24311415). We report human OROV cases in Colombia caused by the novel BR-2015-2024 lineage, which is co-circulating with another previously characterized OROV lineage (*6*). The protocol for this study was approved by the ethics committee of the Corporación para Investigaciones Biológicas (protocol no. SC-6230-1).

## The Study

In early January 2024, health authorities noticed a slight increase in acute febrile cases in Leticia municipality, which has >53,000 inhabitants, in the Amazonas department of Colombia. A total of 117 persons reporting chills (94.6%), headache (87.2%), arthralgia (65%), myalgia (41%), diarrhea (34.2%), fatigue (33.3%), and rash (6%) were treated at the emergency room of a Leticia hospital during a 5-week period. Although fever (>38.5°C) was confirmed at the doctor’s office in >46% of patients, <3% had respiratory symptoms. No hemorrhagic manifestations were observed in the febrile patients, and severe illnesses or hospitalizations were not reported. Patients were 7–92 years of age; 60 were male and 57 female. After obtaining written consent from adults and the children’s parents or legal guardians, hospital staff collected serum samples. 

Frozen serum samples were shipped by air >1,300 km to One Health Colombia in Medellin, Colombia, a center established by the Global Health Institute at the University of Wisconsin, Madison (Madison, WI, USA), and the National University of Colombia-Medellin. According to center guidelines at One Health Colombia (*6*), we tested the serum samples for Zika virus (ZIKV), Mayaro virus (MAYV), chikungunya virus, DENV, OROV, hepatitis B and C viruses, *Leptospira* spp., and *Plasmodium* spp. (Appendix Table 1).

We detected the DENV genome in 8 samples by using the Zika, chikungunya, and dengue virus Trioplex real-time qRT-PCR (Appendix Table 1); 4 samples were positive for the DENV nonstructural protein 1, and 3 samples were positive for DENV IgM. The study population (n = 117) had a high DENV IgG prevalence of 77.7%. All samples were negative for ZIKV, CHIKV, and MAYV RNA. We detected *Plasmodium* spp. DNA in 7 samples by quantitative PCR, and 5 samples were positive for the *P. vivax* antigen. No samples were positive for hepatitis B virus surface antigen, hepatitis C virus antibody, or *Leptospira* spp. DNA.

**Figure 1:**
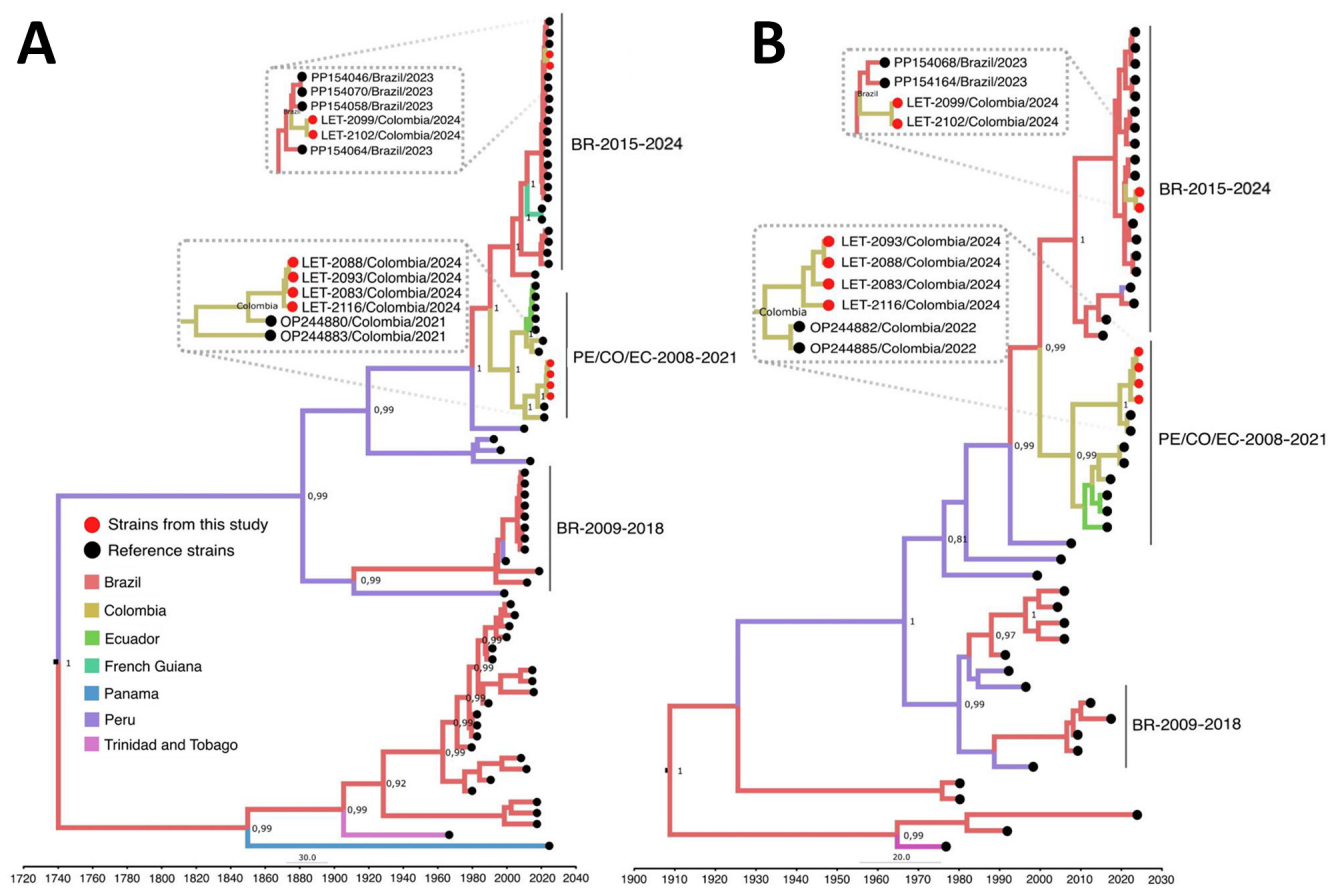
Time-scaled Bayesian phylogeographic analysis of large and small segments of co-circulating Oropouche virus lineages, Amazon Basin, Colombia, 2024. Bayesian phylogenetic trees of large (A) and small (B) gene segments were estimated by using the Bayesian Markov chain Monte Carlo method (>100 million generations) in Beast (https://beast.community) and ModelFinder in IQ-TREE (http://www.iqtree.org) (ultrafast bootstrapping and 1,000 replicates). Red solid circles indicate viruses from this study that begin with LET for Leticia, Colombia. Phylogeny branches are colored according to their descendant place of origin. Best-fit model was selected according to Bayesian information criteria, and a strict molecular clock model was used. Bayesian posterior values (>0.8) are annotated at specific nodes of the trees. Sequences from this study were compared with reference sequences from other studies. Main clusters are indicated by using the following reference labels: BR-2015-2024 cluster represents the recent outbreak of the new OROV lineage in Brazil during 2015–2024; PE/CO/EC-2008-2021 cluster represents sequences from Colombia, Peru, and Ecuador during 2008–2021; and BR-2009-2018 cluster represents sequences from Brazil during 2009–2018. Scale bar indicates nucleotide substitutions per site.

**Figure 2:**
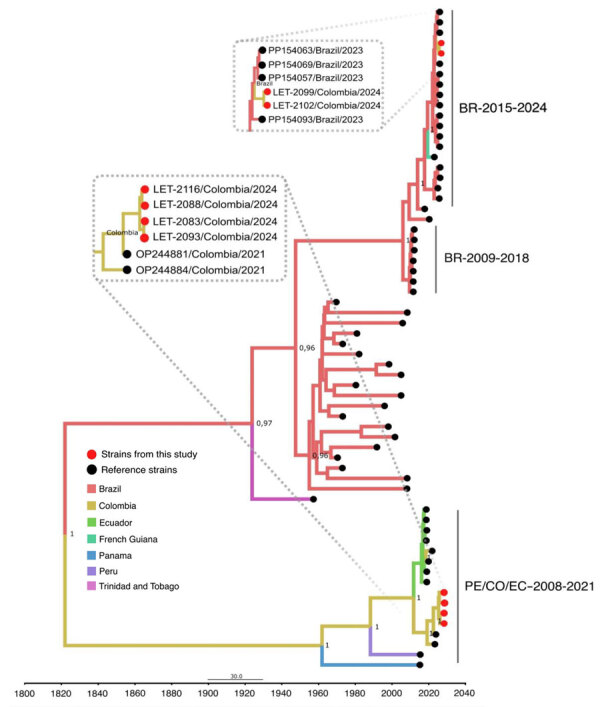
Time-scaled Bayesian phylogeographic analysis of medium segments of co-circulating Oropouche virus lineages, Amazon Basin, Colombia, 2024. Bayesian phylogenetic tree of medium gene segments were estimated by using the Bayesian Markov chain Monte Carlo method (>100 million generations) in Beast (https://beast.community) and ModelFinder in IQ-TREE (http://www.iqtree.org) (ultrafast bootstrapping and 1,000 replicates). Red solid circles indicate viruses from this study that begin with LET for Leticia, Colombia. Phylogeny branches are colored according to their descendant place of origin. Best-fit model was selected according to Bayesian information criteria, and uncorrelated relaxed molecular clock model was used. Bayesian posterior values (>0.8) are annotated at specific nodes of the trees. Sequences from this study were compared with reference sequences from other studies. Main clusters are indicated by using the following reference labels: BR-2015-2024 cluster represents the recent outbreak of the new OROV lineage in Brazil during 2015–2024; PE/CO/EC-2008-2021 cluster represents sequences from Colombia, Peru, and Ecuador during 2008–2021; and BR-2009-2018 cluster represents sequences from Brazil during 2009–2018. Scale bar indicates nucleotide substitutions per site.

**Figure 3:**
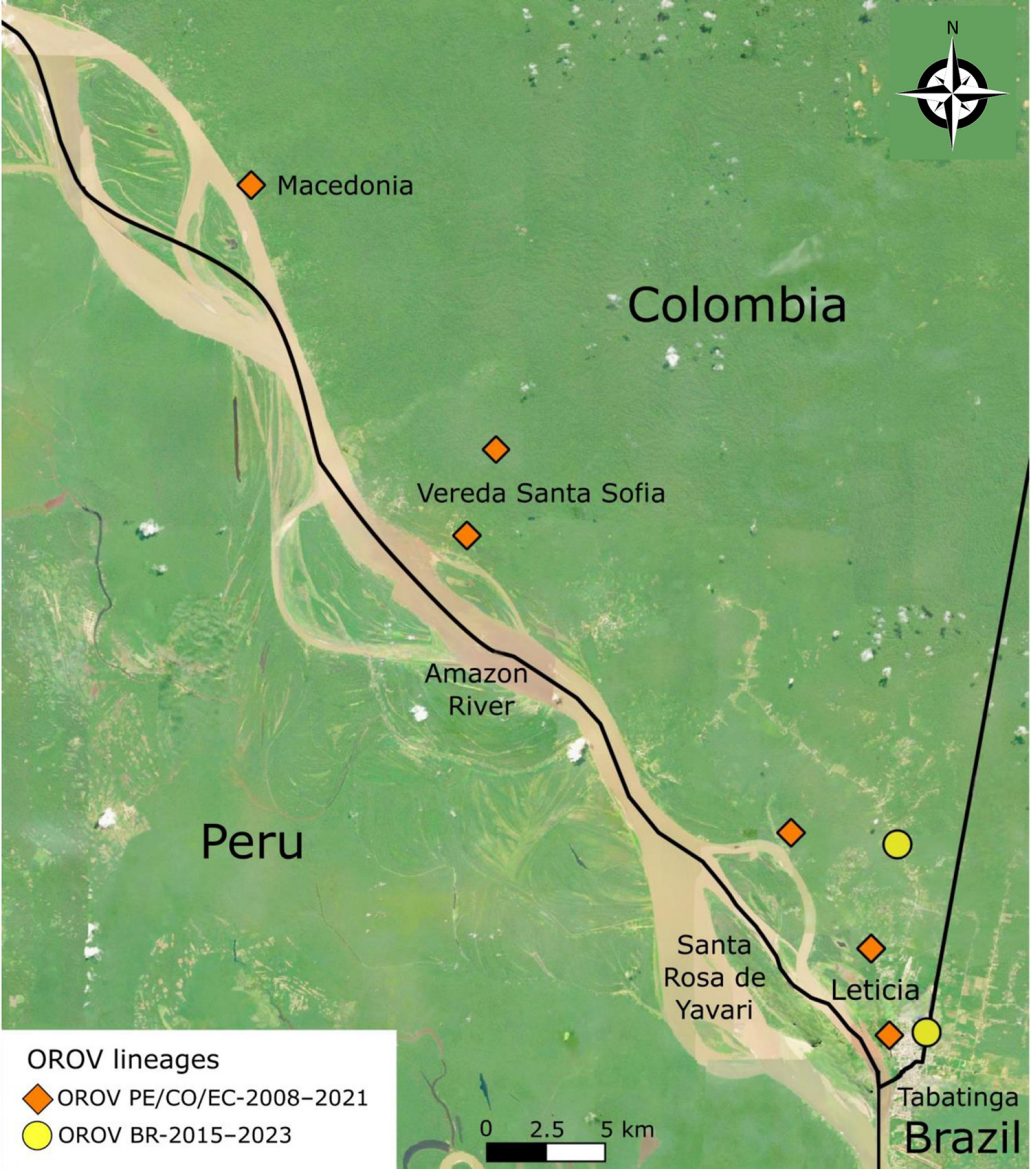
Leticia municipality and Three Borders (Colombia, Peru, and Brazil) region in study of co-circulation of 2 Oropouche virus lineages, Amazon Basin, Colombia, 2024. Symbols indicate residential locations of 8 patients infected with OROV in Leticia municipality. Colored circles and diamonds indicate patients infected with each OROV lineage. Macedonia, Vereda Santa Sofia, Santa Rosa de Yavari, and Tabatinga correspond to indigenous communities or cities. Map was created by using QGIS 3.36.0 RC (https://www.qgis.org). OROV, Oropouche virus.

Using an OROV qRT-PCR designed at One Health Colombia (*6*), we detected OROV L and M segment RNA in 8 samples. We conducted next generation sequencing by using metagenomic and target enrichment approaches for those 8 serum specimens, as described previously (*6*). We conducted phylogenetic analysis of the OROV L, M, and S segments isolated from patient samples by using available OROV genomes and sequences of the new clade from Brazil. OROV BR-2015-2024 from Brazil (*9*; F.C.M. Iani et al., unpub. data) was present in 2 samples from Leticia municipality, designated as LET-2099 and LET-2102 (Figures 1, 2). Although the L and S segments of those 2 OROV samples each branched as paraphyletic clades basal to OROV PE/CO/EC-2008-2021, the M segment sequences were more closely related to OROV BR-2009-2018 sequences. Amino acid changes within lineage BR-2015-2024 indicated multiple mutations in L and M segments when compared with those in ancestral virus strains (Appendix Figure). In addition, in 6 of the 8 samples, we detected the OROV PE/CO/EC-2008-2021 lineage, which circulated in Colombia during 2019–2021 (Table) (*6*). The 8 OROV-positive patients did not exhibit co-infections with the other tested organisms, aside from clinical manifestations of acute undifferentiated fever (Appendix Table 2), and they had not traveled outside of Leticia municipality or its surrounding areas in the 2 weeks before symptom onset (Figure 3). We deposited 6 full-length OROV genomes analyzed in this study, including the 2 novel OROV lineages, into GenBank (accession nos. PP477303–20).

**Table:** Characteristics of 8 human febrile cases of OROV infection in study of co-circulation of 2 Oropouche virus lineages, Amazon Basin, Colombia, 2024*

ID no.	Age, y/sex	Symptom onset date	Sampling date	Ct value	OROV clade
LET-2040	27/M	2024 Jan 16	2024 Jan 19	32.9	PE/CO/EC-2008-2021
LET-2083	23/M	2024 Feb 1	2024 Feb 5	28.6	PE/CO/EC-2008-2021
LET-2088	22/M	2024 Feb 4	2024 Feb 6	31.5	PE/CO/EC-2008-2021
LET-2093	15/F	2024 Feb 5	2024 Feb 8	33.2	PE/CO/EC-2008-2021
LET-2099	24/M	2024 Feb 8	2024 Feb 12	28.7	BR-2015-2024
LET-2102	27/M	2024 Feb 11	2024 Feb 13	30.1	BR-2015-2024
LET-2116	49/F	2024 Feb 13	2024 Feb 15	34.2	PE/CO/EC-2008-2021
LET-2117	66/F	2024 Feb 10	2024 Feb 15	34.4	PE/CO/EC-2008-2021

*OROV was detected in serum samples by using quantitative reverse transcription PCR, and Ct values were determined. Ct, cycle threshold; ID, identification; LET, Leticia; OROV, Oropouche virus.

We report the circulation of the novel orthobunyavirus, OROV BR-2015-2024, in Colombia that has been documented in Brazil. The numerous mutations in OROV BR-2015-2024 RNA-dependent RNA polymerase and glycoproteins likely enhanced replication and immune evasion capabilities, increasing virus fitness and transmission. In addition, we detected co-circulation of OROV PE/CO/EC-2008-2021 lineage (*6*) in 6 patients. The identification of co-circulating strains of OROV exemplifies the evolving nature of orthobunyaviruses and raises concerns about future reassortment events and emergence of new lineages having more severe clinical phenotypes and enhanced vector competence. In an arbovirus hotspot, such as Leticia, it is unknown if cross-protective immunity will exist between the 2 OROV lineages. Previously, a reassortant OROV isolated from outbreaks in Iquitos, the largest city in the Amazon of Peru, provided limited cross-protection against a different OROV strain (*10*).

Leticia municipality, in the southernmost region in Colombia (Figure 3), remains isolated from the rest of Colombia’s road network, and travelers typically reach Leticia by aircraft. Leticia city, the capital of Amazonas department of Colombia, is in Leticia municipality, where the borders of Colombia, Brazil, and Peru converge, forming an area known as the Three Borders. Tabatinga city, in the state of Amazonas in Brazil, is located across from Leticia city, forming a unique suburban area near Santa Rosa de Yavarí, Peru, which is on an island in the Amazon River (*11*). The new OROV lineage is likely spreading rapidly in those borderlands because of the considerable human mobility related to local businesses. Furthermore, we believe that the novel OROV lineage in Leticia came from either a nearby settlement or air travelers from the Amazon in Brazil, where persistent outbreaks are occurring.

## Conclusions

One Health Colombia has previously reported outbreaks of ZIKV (*12*), DENV (*13*), OROV (*6*), and, in 2024, MAYV (*14*) in Colombia, which underscores the dynamic landscape of reemerging arboviruses in the region. The sustained transmission of established strains and the unpredictable reassortment events of OROVs causing outbreaks is yet another demonstration of the public health challenges associated with prevention and control of arbovirus reemergence in South America. Continued surveillance and molecular characterization of OROV and other arboviruses are needed to prevent the spread of arboviral diseases in the Americas.

Appendix Additional information for co-circulation of 2 Oropouche virus lineages, Amazon Basin, Colombia, 2024.
