# Supplementary material for: Co-Circulation of 2 Oropouche Virus Lineages, Amazon Basin, Colombia, 2024
Source: Emerg Infect Dis. 2024 Nov;30(11):2375–80. doi: 10.3201/eid3011.240405 (PMC11521167; doi:10.3201/eid3011.240405)
Supplement: Appendix — Additional information for co-circulation of 2 Oropouche virus lineages, Amazon Basin, Colombia, 2024. [file 24-0405-Techapp-s1.pdf]

Article DOI: https://doi.org/10.3201/eid3011.240405

EID cannot ensure accessibility for supplementary materials supplied by authors. Readers who have difficulty accessing supplementary content should contact the authors for assistance.

Co-Circulation of 2 Oropouche Virus Lineages, Amazon Basin, Colombia, 2024

Appendix

Appendix Table 1. Protocols for microbiologic assays performed at One Health Colombia, 2024*

Assay	Pathogen	Protocol†
Oropouche real-time qRT-PCR	OROV	(*1*)
ZCD Trioplex real-time qRT-PCR	ZIKV, CHIKV, DENV	(*2*)
Mayaro real-time qRT-PCR	MAYV	(*3*)
DENV nonstructural protein 1	DENV	Bioline Dengue Duo (Dengue NS1 Ag + IgG/IgM)
DENV IgM	DENV	Bioline Dengue Duo (Dengue NS1 Ag + IgG/IgM)
DENV IgG	DENV	Bioline Dengue Duo (Dengue NS1 Ag + IgG/IgM)
Hepatitis B virus surface antigen	Hepatitis B virus	ARCHITECT HBsAg Qualitative Reagent Kit
Hepatitis C virus antibody	Hepatitis C virus	ARCHITECT Anti-HCV
Malaria qPCR	*Plasmodium* spp.	(*4*)
Malaria antigen	*P. vivax*	Bioline Malaria Ag P.f/P.v
Leptospira qPCR	*Leptospira* spp.	(*5*)

*CHIKV, chikungunya virus; DENV, dengue virus; MAYV, Mayaro virus; OROV, Oropouche virus; qPCR, quantitative PCR; qRT-PCR, quantitative reverse transcription PCR; RDT, rapid diagnostic test; ZIKV, Zika virus.
†Protocols for assays were obtained from published literature or the indicated commercially available kits from Abbott Laboratories (https://www.abbott.com).

Appendix Table 2. Clinical manifestations of patients infected with each OROV lineage in Leticia municipality, Colombia, 2024*

Symptoms	OROV PE/CO/EC-2008-2021, n = 6	OROV BR-2015-2024, n = 2
Fever	1	1
Headache	6	2
Myalgia	5	2
Arthralgia	6	1
Chills	3	0
Fatigue	5	2
Rash	1	0
Diarrhea	3	1

*Values are number of patients displaying each symptom. OROV, Oropouche virus.

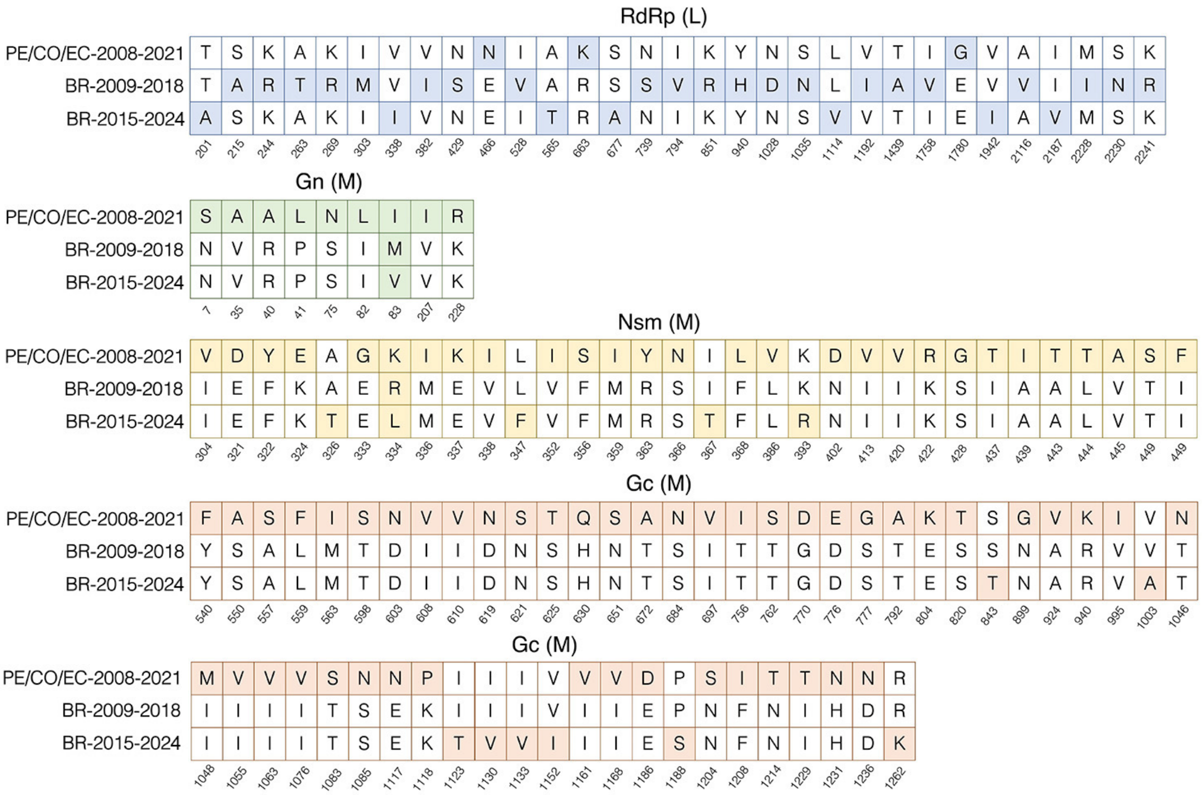

Appendix Figure. Amino acid substitutions in OROV lineages. Amino acid changes are indicated for OROV proteins across 3 major clades: PE/CO/EC-2008-2021, BR-2009-2018, and the novel BR-2015-2024 lineage. A total of 31 changes were observed in the RdRp (L segment), 9 in Gn (M segment), 32 in Nsm (M segment), and 55 in Gc (M segment). No amino acid changes were found in the small segment. Values under squares indicate amino acid position within the specific protein. Representative strains were determined by calculating the consensus sequence from the respective group of sequences within each lineage. Gc, glycoprotein c; Gn, glycoprotein n; L, large; M, medium; Nsm, nonstructural m; RdRp, RNA-dependent RNA polymerase.

References

1. Ciuoderis KA, Berg MG, Perez LJ, Hadji A, Perez-Restrepo LS, Aristizabal LC, et al. Oropouche virus as an emerging cause of acute febrile illness in Colombia. Emerg Microbes Infect. 2022;11:2645–57. PubMed https://doi.org/10.1080/22221751.2022.2136536

2. Waggoner JJ, Gresh L, Mohamed-Hadley A, Ballesteros G, Davila MJV, Tellez Y, et al. Single-reaction multiplex reverse transcription PCR for detection of Zika, chikungunya, and dengue viruses. Emerg Infect Dis. 2016;22:1295–7. PubMed https://doi.org/10.3201/eid2207.160326

3. Waggoner JJ, Rojas A, Mohamed-Hadley A, de Guillén YA, Pinsky BA. Real-time RT-PCR for Mayaro virus detection in plasma and urine. J Clin Virol. 2018; 98:1–4. PubMed https://doi.org/10.1016/j.jcv.2017.11.006

4. Kamau E, Alemayehu S, Feghali KC, Saunders D, Ockenhouse CF. Multiplex qPCR for detection and absolute quantification of malaria. PLoS One. 2013;8:e71539. PubMed https://doi.org/10.1371/journal.pone.0071539

5. Waggoner JJ, Abeynayake J, Balassiano I, Lefterova M, Sahoo MK, Liu Y, et al. Multiplex nucleic acid amplification test for diagnosis of dengue fever, malaria, and leptospirosis. J Clin Microbiol. 2014;52:2011–8. PubMed https://doi.org/10.1128/JCM.00341-14
